# Seasonal Variation in Whole‐Body Elemental Concentrations and Excreta Production of a Riparian Orb‐Weaving Spider ( *Larinioides cornutus* )

**DOI:** 10.1002/ece3.74385

**Published:** 2026-09-26

**Authors:** Colton Herzog, Shawn M. Wilder

**Affiliations:** ^1^ Department of Biology Oklahoma State University Stillwater Oklahoma USA

**Keywords:** ecological stoichiometry, ionomics, orb‐weaving spiders, temporal nutrient cycling, trace elements

## Abstract

Predators influence nutrient cycling by assimilating elements from their prey and returning nutrients to ecosystems via excretion and discarded prey remains. However, seasonal variation in the contribution of predators to nutrient cycling remains poorly understood, particularly for terrestrial arthropod predators such as spiders. We examined temporal variation in whole‐body elemental concentrations, excreta C and N concentrations, and cumulative excreta output in a riparian orb‐weaving spider ( *Larinioides cornutus* ) across the growing season of 2024. Adult females were collected across seven sampling periods spanning mid‐June to early October, and whole‐body concentrations of macronutrients (carbon [C], nitrogen [N], phosphorus [P]) and multiple trace elements were quantified. Whole‐body N and P concentrations, and several trace elements (potassium [K], lithium [Li], manganese [Mn], sodium [Na], nickel [Ni], sulfur [S], silicon [Si], and strontium [Sr]) exhibited significant seasonal variation, with elements differing in both the direction and shape of temporal patterns. Cumulative excreta production also varied significantly across the season and exhibited a mid‐season peak, whereas excreta C and N concentrations remained comparatively stable. Model selection favored linear relationships for most responses, although quadratic models were supported for some variables (i.e., spider body mass, excreta production, K concentrations, and Na concentrations), indicating non‐monotonic seasonal dynamics. These findings suggest that seasonal shifts in prey availability and/or spider physiology influence nutrient assimilation and excreta production in riparian spiders. Our results highlight the importance of incorporating both trace elements and seasonal context when evaluating predator‐mediated nutrient cycling in terrestrial ecosystems.

## Graphical abstract

Predators play an important role in ecosystem nutrient cycling, yet seasonal variation in predator‐mediated nutrient cycles remains poorly understood. We show that the whole‐body elemental composition and excreta production of the riparian orb‐weaving spider, *Larinioides cornutus* , vary substantially across the growing season, indicating that predator‐mediated nutrient retention and release are temporally dynamic. These findings highlight the importance of incorporating seasonal context and trace elements when evaluating nutrient cycles in terrestrial ecosystems.

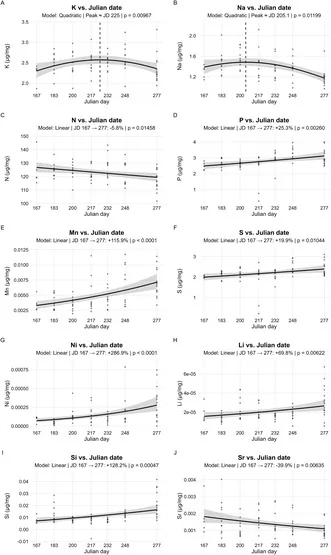

## 1 Introduction

Predator‐driven nutrient cycling can strongly influence biogeochemical processes and ecosystem functioning (Kitchell et al. 1979; Elser and Urabe 1999; Vanni 2002; Vanni et al. 2002; Fagan et al. 2002; Schmitz et al. 2010, 2014, 2018). In predators, this occurs when: (1) nutrients acquired from prey are returned to the environment as excreta or discarded prey remains, (2) predators themselves become nutrient sources for higher trophic levels when consumed, or (3) predator mortality transfers nutrients into detrital pathways (Schmitz et al. 2010). Prominent examples of these mechanisms include wolves generating landscape heterogeneity through the spatial distribution of prey carcasses (Bump et al. 2009), coastal seabirds transporting marine‐derived nutrients onto land and subsidizing terrestrial food webs (Barret et al. 2005), and brown bears contributing substantial inputs of salmon‐derived nitrogen to riparian ecosystems (Hilderbrand et al. 1999).

Although these examples demonstrate that predators can act as powerful drivers of nutrient redistribution, the magnitude and direction of their effects are not uniform (Schmitz et al. 2010; Kaspari 2021). Predator contributions to nutrient cycling depend on consumer physiology, diet composition, and the balance between nutrient assimilation and excretion, all of which determine the amounts and ratios of nutrients contributed to ecosystems by predators (Elser and Urabe 1999; Fagan et al. 2002; Sterner and Elser 2002; Schmitz et al. 2010). These processes may be especially important for invertebrate predators, which often occur at high densities and exhibit rapid turnover in both individuals and communities (Sagi and Hawlena 2021). Yet, despite their numerical dominance in many ecosystems, the stoichiometric roles of invertebrate predators remain comparatively understudied relative to vertebrate consumers.

Among invertebrate predators, spiders are particularly prominent. Collectively, spiders consume an estimated 400–800 million tons of prey biomass annually and occupy all terrestrial systems except for Antarctica (Nyffeler and Birkhofer 2017). Empirical studies demonstrate that spider‐mediated nutrient inputs can alter ecosystem processes across a wide range of systems. As examples: in the arctic tundra, linyphiid spiders disperse nutrient‐rich waste early in the growing season, modifying soil nutrient availability for lower trophic levels (Hodkinson et al. 2001); in tropical forests, salticid spiders deposit nutrients directly onto host bromeliads, enhancing plant growth (Romero et al. 2006); and across desert, grassland, tropical, and urban habitats, intra‐ and interspecific variation in spider stoichiometry has been shown to influence whole‐body elemental composition, subsequently altering the quantity and quality of nutrients transferred through food webs (Ludwig et al. 2018; Trubl and Johnson 2019; Sobcyzk et al. 2020; Herzog et al. 2025). Together, these studies indicate that spider contributions to ecosystem nutrient dynamics are context dependent rather than fixed, varying with ecological conditions and resource availability (Hawlena et al. 2012; Filipiak and Weiner 2017; Leal et al. 2017; Filipiak and Filipiak 2022; Herzog et al. 2023).

Given the large amount of prey biomass that spiders consume annually, shifts in the prey they encounter could have consequences for nutrient dynamics (Nyffeler and Birkhofer 2017). Invertebrate abundance and community composition often change seasonally (Harrison et al. 2005; Yamarnura et al. 2006; Dennis et al. 2021), altering resources available to spiders. Such changes may influence not only the amount of biomass spiders assimilate into new tissues, but also the amount and composition of nutrients they release as waste (Schmitz et al. 2010, 2018; Filipiak 2016; Kaspari 2021; Filipiak and Filipiak 2022). However, the extent to which seasonal variation in resource availability alters predator stoichiometry—particularly the balance between elemental assimilation into body tissues vs. release as waste products—remains poorly understood.

One mechanism by which temporal variation in resource availability may shape predator‐mediated nutrient cycling is through predator physiology. The fate of ingested elements depends on whether predators retain them for structural or metabolic functions, or instead excrete them as waste. Assimilation strategies differ among elements: some are preferentially retained or bioaccumulated (Hendrickx et al. 2003; Jung and Lee 2012; Herzog et al. 2023, 2025), whereas others are more readily excreted, making predators either nutrient sources or sinks depending on the element and ecological context (Hodkinson et al. 2001; Romero et al. 2006; Hawlena et al. 2012; Filipiak et al. 2021; Wilder, Barnes, et al. 2025; Wilder, Herzog, et al. 2025). Seasonal variation in reproductive output may also influence the amounts and types of nutrients that female predators assimilate versus excrete. For generalist predators like spiders, seasonal variation in dietary intake and requirements may therefore drive physiological changes in nutrient uptake, storage, or waste production (Elser and Urabe 1999; Fagan et al. 2002; Sterner and Elser 2002; Evans‐White et al. 2005; Kaspari 2021). Yet, despite growing evidence that spider‐mediated nutrient inputs are context dependent, it remains unclear how shifts in prey availability influence predator whole‐body elemental concentrations, and whether these changes are expressed through elemental retention in body tissues or released to the environment as excreta.

In this study, we investigated seasonal patterns in body mass, whole‐body elemental concentrations, excreta C and N concentrations, and cumulative excreta production in a riparian orb‐weaving spider ( *Larinioides cornutus* ). We predicted that spider body mass and excreta mass would vary across the season, reflecting shifts in resource availability and predator physiology. We further predicted that whole‐body elemental concentrations (C, N, P, and trace elements) and excreta C and N concentrations would vary throughout the sampling season. Finally, because the physiological roles and dietary requirements of many trace elements in spiders remain poorly understood, patterns in trace‐element concentrations were treated as exploratory, providing insight into temporal variation in elemental accumulation within spider bodies.

## 2 Methods

### 2.1 Study Species

From June to the start of October, we collected 20 adult female *Larinioides cornutus* per sampling period from riparian vegetation along the banks of Lake McMurtry in Noble County, Oklahoma. This sampling schedule resulted in seven collection trials, corresponding to Julian days 167, 183, 200, 217, 232, 248, and 277 (i.e., June 15th, July 1st, July 18th, August 4th, August 19th, September 4th, October 3rd respectively). In the laboratory, spiders were housed individually for 7 days in clear plastic containers (7.4 cm height × 16.2 cm diameter) under controlled environmental conditions (25°C ± 1°C; 14L:10D) and provided water ad libitum. Collections ceased in October when adult populations in the field had noticeably declined.

### 2.2 Spider Whole‐Body Sample Collection

For each sampling trial, a subset of collected spiders was randomly selected for whole‐body elemental analysis (after the 7‐day in‐lab housing period). Ten individuals per trial were typically analyzed; however, only five individuals from the first June trial were included due to sample loss during elemental processing. In contrast, 15 individuals from the final October trial were analyzed because spiders were visibly smaller, and additional individuals were collected to ensure sufficient biomass for multiple elemental analyses (across all trials total *n* = 70). Following collection, all spiders were held for 7 days to allow gut clearance, after which individuals that were randomly chosen for whole‐body analysis were frozen, while the rest were released at sites separate from the original collection location. Sacrificed specimens were dried at 60°C for 72 h, weighed to obtain whole‐body dry mass, and homogenized with a ball‐bearing mixer. Dried whole‐body samples were stored until subsequent C, N, and trace element analyses were conducted.

### 2.3 Spider Excretion Sample Collection

Excreta was collected from all spiders in every trial, with each of the 20 individuals per trial producing excreta; however, samples were pooled post‐collection to achieve sufficient mass for C and N analysis (final *n* = 50 after pooling; see elemental concentration analysis below). Spider excretion is typically deposited within 72 h after individuals are brought into the laboratory (Barnes 2010; Herzog et al. 2023, 2025). However, we observed excreta production from some spiders after 72 h. Thus, to ensure complete excreta collection, spiders were held for 7 days to allow for complete gut clearance. Following collection, each excreta sample was dissolved in 1.5–2.0 mL of distilled water and transferred to 2 mL centrifuge tubes. Samples were centrifuged at 13,000 rpm for 10 min to form a pellet and then dried at 60°C for 48 h to fully evaporate the water; no liquid was removed prior to drying. Dried excreta samples were stored for C and N analysis.

### 2.4 Elemental Concentrations Analyses

We measured elemental concentrations in spider excreta and whole bodies. Although we initially attempted to quantify a broader suite of elements in spider excreta, insufficient sample mass and analytical limitations prevented reliable measurements for P and trace elements. As a result, excreta samples were processed for C and N analysis only. Individual excreta samples weighing < 3 mg were randomly pooled with other low‐mass excreta samples from different spiders within the same trial date to obtain sufficient material for analysis (excreta *n* = 2–13 samples per trial after pooling). After pooling low‐mass samples within trials, a total of 50 excreta samples were analyzed for C and N across all sampling periods.

To evaluate whether pooling low‐mass excreta samples influenced variability, multivariate structure, or downstream statistical inference, we compared pooled and unpooled excreta samples using univariate, multivariate, and model‐based approaches. Variance comparisons indicated no difference in variability between pooled and unpooled samples for either C or N (Levene's tests; *p* > 0.74 for both). A PERMDISP analysis based on Aitchison (1986) distances from centered log‐ratio transformed C and N data further showed no difference in multivariate dispersion between pooled and unpooled samples (*p* = 0.59). Principal component analysis revealed no effect of pooling on multivariate position, with pooled and unpooled samples not differing along either PC1 (*p* = 0.47) or PC2 (*p* = 0.22), and a PERMANOVA indicated that pooling did not significantly influence overall excreta C and N composition (*p* = 0.46). Finally, generalized linear models fit with and without pooling status as a predictor showed that pooling did not consistently improve model fit or alter inference for C or N.

Whole‐body subsamples and excreta samples were sent to the University of Florida Stable Isotope Laboratory for C and N measurements. Since whole‐body samples always exceeded analytical mass requirements, pooling was not required for whole‐body elemental analyses. Concentrations of other elements in whole bodies were analyzed using inductively coupled plasma–optical emission spectrometry (ICP‐OES; Thermo Scientific iCAP 7400) in the Department of Biology at Oklahoma State University (B, Ba, Ca, Cr, Cu, Fe, K, Li, Mg, Mn, Na, Ni, P, S, Si, Sr, and Zn). ICP‐OES sample preparation followed methods from Prater et al. (2020).

## 3 Data Analysis

### 3.1 Statistical Analyses

All inferential analyses were conducted using generalized linear models (GLMs) with Julian day treated as a continuous predictor. Since response variables (e.g., spider body mass, cumulative excreta production, and whole‐body elemental concentrations, and excreta C and N concentrations) were strictly positive and right‐skewed, models were fit using a Gamma error distribution with a log link. For each response variable, linear (Julian day) and quadratic (Julian day + Julian day^2^) models were evaluated, and model selection was based on Akaike's Information Criterion (AIC) (Akaike 1974). Quadratic models were retained only when their AIC was more than 2 units lower than the corresponding linear model $\left({\mathrm{AIC}}_{\text{quadratic}}-{\mathrm{AIC}}_{\text{linear}}<-2\right)$; otherwise, linear models were selected. Statistical significance of seasonal patterns was assessed using Wald tests of the Julian day term in the selected model. Model predictions with 95% confidence intervals were generated on the link scale and back‐transformed to the response scale for visualization. All analyses were conducted in R (R Core Team).

### 3.2 Spider and Excreta Weight Analyses

**FIGURE 1:**
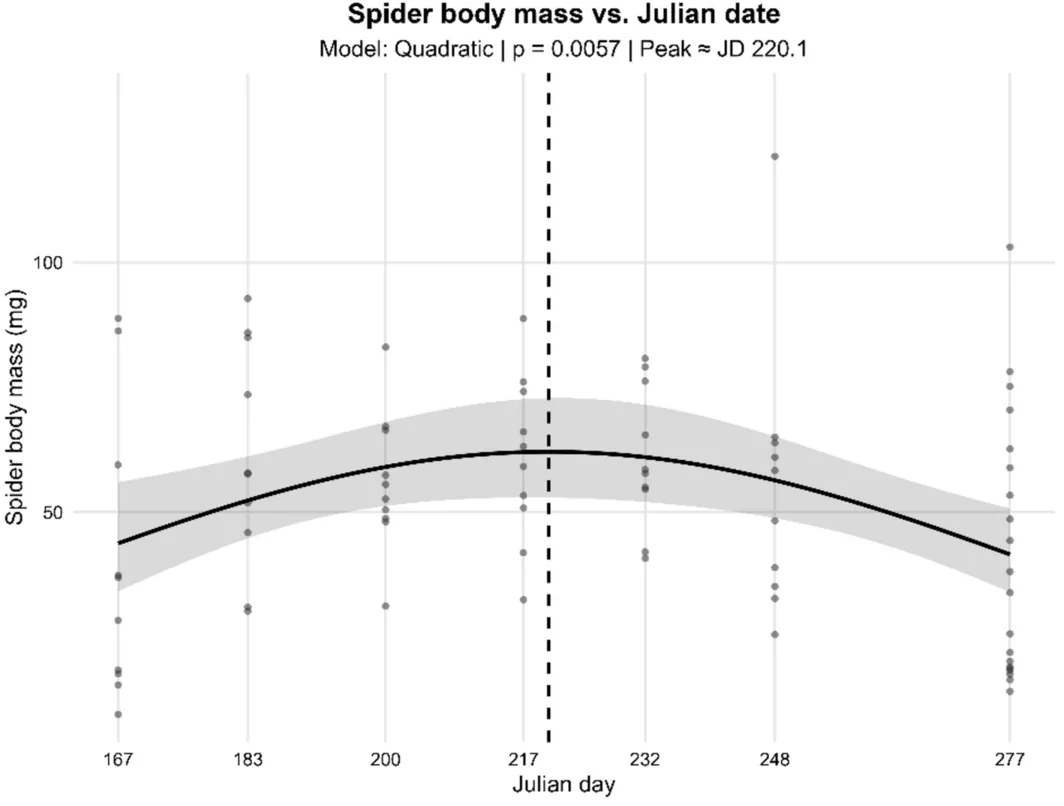
Individual spider dry mass (mg) plotted against Julian day across the sampling season (Days 167–277). Points represent individual spiders. Lines and shaded bands show the fitted GLM (Gamma error distribution with a log link) and its 95% confidence interval. A quadratic model was selected based on AIC, with the dashed vertical line indicating the Julian day of peak predicted body mass.

**FIGURE 2:**
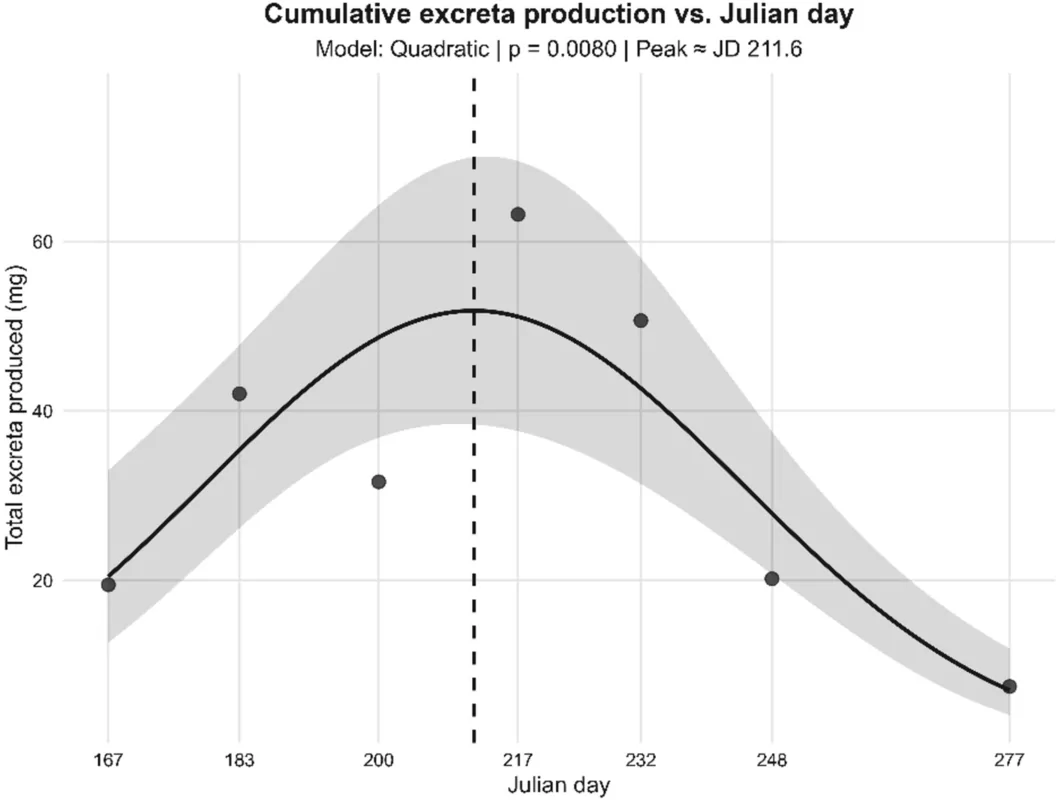
Total pooled excreta mass (mg) produced by spiders across Julian days during the 2024 growing season. Excreta production was calculated by summing all excreta collected within each sampling trial, and trends were analyzed using a quadratic Gamma(log) generalized linear model. Points represent total excreta mass produced by all 20 spiders for each sampling period (Julian day), and the fitted line represents model‐predicted values across Julian day. Shaded regions indicate 95% confidence intervals. The dashed vertical line indicates Julian day corresponding to the model‐predicted peak excreta response.

Spider body mass and excreta production were analyzed within the statistical framework described above. Individual spider dry mass (mg) was modeled as a function of the Julian day corresponding to each trial (Figure 1). When quadratic models were selected, the Julian day corresponding to the peak predicted response was calculated analytically from model coefficients. Total excreta mass per sampling period was calculated by summing excreta samples within each Julian day and analyzed as a response variable using the same regression framework (Figure 2). This analysis reflects temporal variation in aggregate excreta production across sampling periods rather than individual‐level excreta production.

### 3.3 Seasonal Analyses of Whole‐Body and Excreta Elemental Concentrations

Whole‐body elemental concentrations and excreta C and N concentrations were evaluated using the statistical framework described above, with each element analyzed separately (B, Ba, C, Ca, Cr, Cu, Fe, K, Li, Mg, Mn, N, Na, Ni, P, S, Si, Sr, and Zn). Seasonal changes in elemental concentrations were summarized using fitted values generated from the selected regression models across Julian day. *p*‐values associated with the Julian day term in the selected model were used to assess the significance of patterns.

## 4 Results

### 4.1 Seasonal Patterns in Spider Body Mass and Excreta Production

Adult female spider body mass varied significantly across the sampling period (Julian days 167–277; Figure 1; *p* = 0.006). A quadratic model was selected over a linear model based on AIC, indicating non‐linear seasonal variation in body mass (Table 1). Predicted spider dry mass increased from early summer to mid‐season and declined toward the end of the sampling period, with peak body mass occurring at Julian day 220 (August 7th).

**TABLE 1:** Seasonal patterns in spider body mass, excreta mass, and elemental concentrations were assessed using generalized linear models (GLMs) with a Gamma error distribution and log link, with Julian day treated as a continuous predictor.

Response variable	*n*	Chosen model	% change	*p*	Sig	ΔAIC
Spider Body Mass	80	**Quadratic**	—	**0.006**	**	**−6.06**
Excreta Production	7	**Quadratic**	—	**0.008**	**	**−11.27**
C (excreta)	50	Linear	6.4	0.131	ns	1.93
N (excreta)	50	Linear	10.2	0.308	ns	1.17
B (whole body)	70	Linear	−9.8	0.753	ns	1.59
Ba (whole body)	70	Linear	51.2	0.071	ns	1.28
C (whole body)	70	Linear	1.35	0.462	ns	1.99
Ca (whole body)	70	Linear	16.7	0.208	ns	0.96
Cr (whole body)	70	Quadratic	—	0.091	ns	−4.56
Cu (whole body)	70	Linear	−15.4	0.175	ns	0.17
Fe (whole body)	70	Linear	−14.6	0.419	ns	1.75
K (whole body)	70	**Quadratic**	**__**	**0.01**	**	**−5.22**
Li (whole body)	70	**Linear**	**69.8**	**0.006**	**	**−0.275**
Mg (whole body)	70	Quadratic	—	0.091	ns	−2.17
Mn (whole body)	70	**Linear**	**116**	**0.00001**	***	**1.9**
N (whole body)	70	**Linear**	**−5.77**	**0.015**	*	**−1.11**
Na (whole body)	70	**Quadratic**	—	**0.012**	*	**−6.22**
Ni (whole body)	70	**Linear**	**287**	**0.00004**	***	**1.98**
P (whole body)	70	**Linear**	**25.3**	**0.003**	**	**1.98**
S (whole body)	70	**Linear**	**19.9**	**0.01**	*	**1.6**
Si (whole body)	70	**Linear**	**128**	**0.0005**	***	**−0.07**
Sr (whole body)	70	**Linear**	**−39.9**	**0.006**	**	**1.26**
Zn (whole body)	70	Linear	5.8	0.573	ns	1.4

*Note:* For each response variable, linear and quadratic models were compared using Akaike's Information Criterion (AIC). Quadratic models were retained only when their AIC was more than 2 units lower than the corresponding linear model $\left({\mathrm{AIC}}_{\text{quadratic}}-{\mathrm{AIC}}_{\text{linear}}<-2\right)$. For responses best described by linear models, percent change represents the difference between model‐predicted values at the first and last Julian days sampled (167–277). Percent change was not calculated for responses best described by quadratic models because seasonal patterns were non‐monotonic. Significance codes for bolded numbers: *p* < 0.05 (*), *p* < 0.01 (**), *p* < 0.001 (***); ns = not significant.

Total excreta production also varied significantly across sampling periods (Figure 2; *p* = 0.008). Cumulative excreta output peaked at Julian day 212 (July 30th) and was lowest during early and late sampling periods, reflecting temporal variation in cumulative excreta output.

### 4.2 Seasonal Patterns in Excreta C and N Concentrations

C and N concentrations in spider excreta showed no significant temporal patterns across the sampling season (Figures S1 and S2; *p* > 0.05). Although model predictions indicated slight increases in excreta C and N concentrations from Julian day 167 to 277, these changes were small (< 11%) and not statistically significant.

### 4.3 Seasonal Patterns in Whole‐Body Elemental Concentrations

**FIGURE 3:**
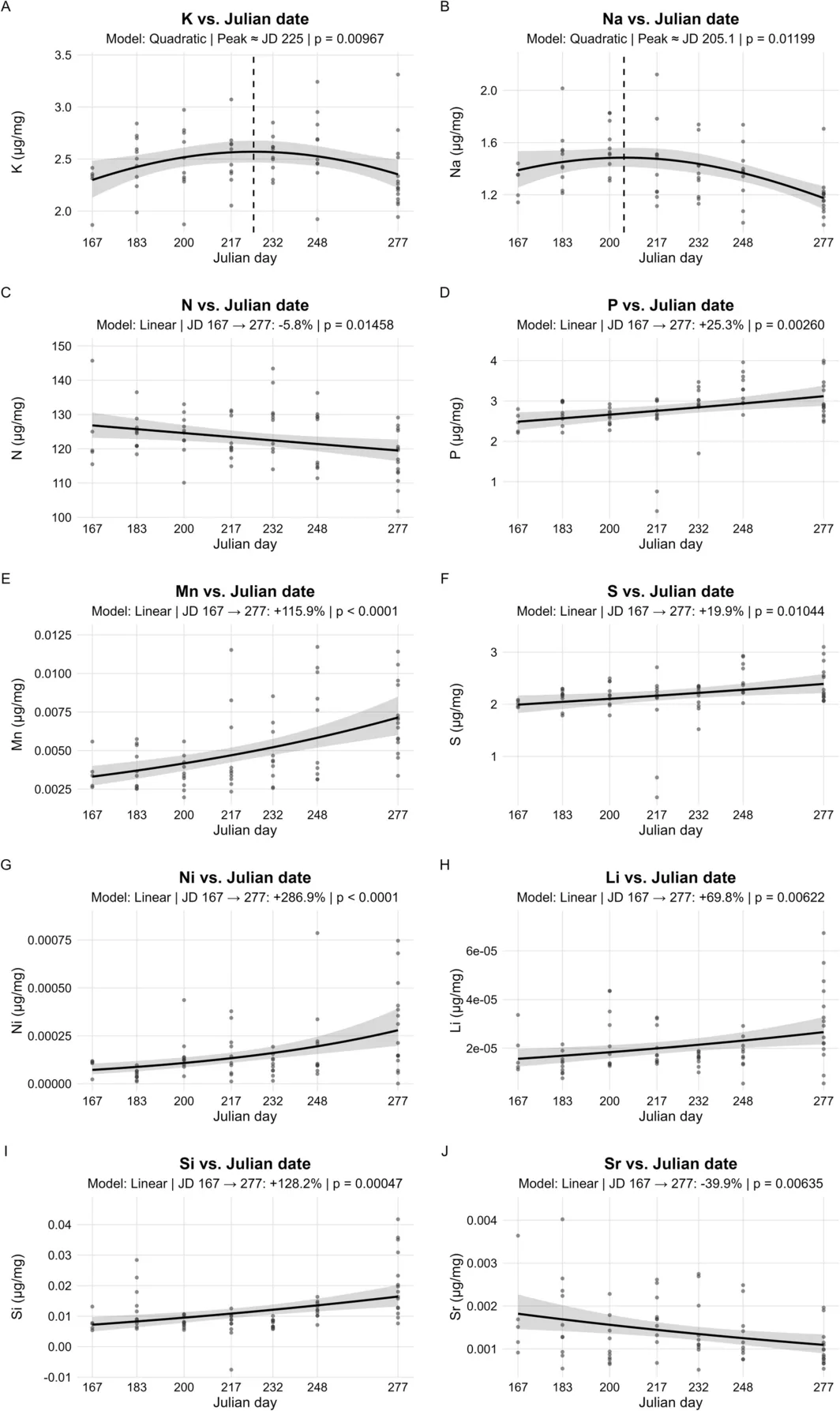
Significant seasonal responses in whole‐body elemental concentrations of adult female *L. cornutus* during 2024. Points represent individual spiders collected on each Julian day. Solid lines show fitted generalized linear models (GLMs; Gamma distribution with log link), and shaded ribbons indicate 95% confidence intervals. Julian days correspond to seven collection trials from mid‐June through early October. Dashed vertical lines denote the predicted peak concentration for elements described by quadratic models.

Whole‐body elemental concentrations exhibited significant temporal variation for several elements (Figure 3; Table 1). In contrast to excreta, whole‐body N concentrations decreased significantly with Julian day (−5.77%, Figure 3C; *p* = 0.015), whereas whole‐body C concentrations remained relatively constant throughout the sampling season (Figure S5; *p* > 0.05). Additionally, whole‐body P concentrations increased during the season by 25.3% (Figure 3D; *p* = 0.003), indicating that some, but not all, macronutrients in *L. cornutus* exhibited seasonal variation.

Beyond macronutrients, eight trace elements—K, Li, Mn, Na, Ni, S, Si, and Sr—showed significant temporal patterns in whole‐body concentrations (Figure 3; Table 1). Model selection based on AIC supported linear seasonal patterns for Li, Mn, Ni, S, Si, and Sr, and quadratic patterns for K and Na. Among trace elements exhibiting linear patterns, Li, Mn, Ni, S, and Si increased across the season, whereas Sr decreased. Furthermore, although AIC supported quadratic models for Cr and Mg, trends for these elements were not statistically significant (Figures S7 and S10). All other elements showed no significant seasonal relationships (Figures S3, S4, S6, S8, S9, and S11).

## 5 Discussion

Our results supported our hypothesis that *L. cornutus* body mass and excreta production would vary seasonally. Both variables showed unimodal responses that peaked mid‐season within 8 days of one another. We also found support for seasonal changes in spider whole‐body (C, N, P, and trace elements) and excreta (C and N) elemental concentrations. While there was stability in excreta macro‐elemental concentrations, seasonal variation was evident in a variety of whole‐body elemental concentrations. Two macronutrients (N and P) and eight trace elements (K, Li, Mn, Na, Ni, S, Si, and Sr) exhibited seasonal patterns in whole‐body elemental concentrations (Figure 3; Table 1). Furthermore, model selection indicated linear seasonal trajectories for Li, Mn, Ni, S, Si, and Sr, whereas K and Na followed quadratic patterns, suggesting non‐monotonic seasonal changes for some variables (Figures 1, 2, 3; Table 1). Hence, our results support the overall idea that the contribution of *L. cornutus* to nutrient cycling can vary across the season.

Since macronutrients play central roles in both organismal physiology and ecosystem processes, shifts in whole‐body N and P concentrations may have important functional implications, especially for predators occupying intermediate terrestrial trophic levels such as spiders (Elser et al. 1996; Elser and Urabe 1999; Fagan et al. 2002; Sterner and Elser 2002; Schmitz et al. 2010, 2018). Notably, these macronutrients exhibited opposing trajectories, with whole‐body N concentrations decreasing by 5.77% and P whole‐body concentrations increasing by 25.3% over the sampling period (Figure 3C,D; Table 1). As a result, predation or mortality of *L. cornutus* at different times during the growing season may influence patterns of macronutrient transfer to upper or lower trophic levels. However, whether similar temporal shifts in whole‐body macronutrient content occur in other invertebrate predators warrants further investigation.

Among the trace elements exhibiting significant temporal variation in *L. cornutus* , Mn, Na, K, S, and Sr represent a subset that have been more extensively examined in arthropod physiology. Na and K are essential for neuromuscular function, ion balance, and osmotic regulation, and their availability can influence consumer performance, behavior, and survival (Welti et al. 2019; Kaspari et al. 2021; Filipiak and Filipiak 2022). Seasonal variation in whole‐body Na and K concentrations may therefore reflect changes in predator dietary composition or physiological demand, particularly given shifting prey communities (Reeves et al. 2021, 2023). Notably, Na and K concentrations followed non‐monotonic trajectories, with peak values occurring during mid‐season (JD ~220 for spider body mass, ~205 for K, and ~225 for Na; Figure 3A,B; Table 1). The temporal alignment of these peaks suggests that changes in spider body condition and whole‐body trace element concentrations may be influenced by shared seasonal factors, such as prey availability or physiological demand. Although whether these elements covary with body mass independent of seasonal timing remains unclear.

Within this same group of commonly studied elements, Mn, S, and Sr are associated with structural and metabolic processes in invertebrates. S plays a central role in protein structure, stability, and function (Ingenbleek and Young 2004; Marelja et al. 2018), while Sr, a Ca analog, can be incorporated into mineralized arthropod tissues such as the exoskeleton (Brannon and Rao 1979; Peters et al. 2008; Katsikini 2016). Moreover, Mn is a critical micronutrient involved in enzymatic processes and oxidative stress regulation (Baden and Eriksson 2006; Jensen and Jensen 2014; Pankau et al. 2022). Patterns in Mn, S, and Sr concentrations suggest that spiders may experience temporally dynamic exposure or assimilation of these elements, potentially mediated by changes in nutrient availability within their habitat (Figure 3E,F,J; Table 1). Collectively, variation in these trace elements indicates that spiders may function as temporally variable reservoirs of biologically important micronutrients, which could influence both the timing and magnitude of elemental transfer through food webs.

Beyond the more commonly examined elements, Li, Si, and Ni remain comparatively less well studied in terrestrial arthropods despite growing evidence that they can accumulate in invertebrate tissues and contribute to trace element cycling within ecosystems (Kaspari 2021; Herzog et al. 2023, 2025). Although Li is not considered an essential micronutrient for arthropods, it can be incorporated into invertebrate tissues through dietary and environmental exposure and appears to exhibit limited homeostatic regulation, which may make predator tissue concentrations sensitive to environmental availability (Bozich et al. 2019; DeBenedictis et al. 2020; Thibon et al. 2021, 2023; Chen et al. 2024). Similarly, Si, while not generally regarded as essential for most arthropods, may enter spider tissues indirectly through plant‐ or detritus‐based food webs via prey consumption or contact with organic matter (Hodkinson et al. 2001; Mwangi et al. 2011; Schaller and Struyf 2013; Nyffeler et al. 2016; Stojanowska et al. 2020; Bartz et al. 2021). Thus, the observed 69.8% and 128% increase in Li and Si whole‐body concentrations suggests that spiders integrate temporal variation in trace element availability through diet and exposure rather than maintaining strict elemental homeostasis (Figure 3H,I; Table 1). Among these elements, Ni is particularly noteworthy due to its strong propensity for bioaccumulation and potential physiological consequences, warranting closer consideration.

Although Ni may function as a micronutrient at low levels, it readily accumulates in arthropod tissues and may be transferred through trophic levels via predation or mortality. The pronounced 287% increase in whole‐body Ni concentrations observed in *L. cornutus* indicates that spiders may function as prominent transient sinks or vectors for Ni in food webs (Figure 3G; Table 1). Similar patterns of Ni accumulation have been documented in other spider taxa, indicating that heavy metal assimilation may be a common feature of predatory arthropods (Hendrickx et al. 2003; Peterson et al. 2003; Jung and Lee 2012; Conti et al. 2018; Beaubien et al. 2020; Stojanowska et al. 2020; Herzog et al. 2025). Together, these accumulation dynamics highlight the potential for invertebrate predators to influence trace metal distribution not only through excretion but also through biomass‐mediated pathways associated with predation and mortality. Whether comparable seasonal patterns of trace element accumulation occur in other predatory invertebrate taxa remains an open question. Furthermore, the physiological impacts of Ni assimilation for predators of spiders, including other spiders, need further investigation.

Taken together, these findings suggest that the contribution of spiders to upward and downward nutrient transfer may vary seasonally within terrestrial food webs. In addition to returning nutrients to lower trophic levels through excretion and mortality, spiders also transfer nutrients upward through predation, as they are consumed by a wide range of vertebrate and invertebrate predators, including other spiders (Gunnarsson 2007; Foelix 2011; Nentwig 2012). Consequently, spiders represent nutrient‐rich biomass capable of transferring both macronutrients and trace elements through trophic interactions. Macronutrients such as C, N, and P contribute to energy content and tissue construction, whereas trace elements such as Fe, Zn, and Ca play important structural and catalytic roles in consumer physiology (Elser et al. 1996; Elser and Urabe 1999; Fagan et al. 2002; Sterner and Elser 2002; Elser et al. 2003; Elser et al. 2016; Zhou and Declerck 2019; Zhang et al. 2023). Quantifying how predator elemental phenotypes change through time is therefore critical for understanding seasonal dynamics in predator‐mediated nutrient cycling.

The physiological mechanisms responsible for these seasonal changes remain unclear. The extent to which these elements are retained within spider biomass versus returned to the environment likely depends on physiological processes governing assimilation, allocation, and storage, which may shift seasonally with changes in growth, reproduction, and prey availability. For example, spiders may prioritize somatic growth earlier in the season, whereas later‐season reproductive investment may alter elemental demands and whole‐body composition, particularly when eggs differ compositionally from female somatic tissues and represent a substantial proportion of total biomass (Foelix 2011; Nentwig 2012; Kleinteich et al. 2014; Wilder and Schneider 2017; Wiggins and Wilder 2022; Herzog et al. 2025). Further work examining how reproduction influences nutrient dynamics may be useful.

Overall, this study demonstrates that seasonal dynamics in predator whole‐body elemental concentrations can be pronounced. Across the growing season, *L. cornutus* exhibited substantial shifts in whole‐body elemental concentrations, including opposing trajectories in N and P and marked increases in several trace elements. For example, whole‐body Ni concentrations increased by 287% over the sampling period, underscoring the extent to which trace metals can accumulate within predator biomass across ecological timescales. Moreover, seasonal shifts in trace element concentrations raise important questions about the physiological consequences of consuming prey that have bioaccumulated trace metals, particularly for predators occupying intermediate trophic levels such as spiders. Therefore, integrating seasonal stoichiometry with physiological and toxicological perspectives will be critical for understanding how predator populations influence the distribution, transfer, and potential biological effects of micronutrient transfer through trophic levels.

## Author Contributions

**Colton Herzog:** conceptualization (equal), data curation (equal), formal analysis (equal), investigation (equal), methodology (equal), resources (equal), validation (equal), visualization (equal), writing – original draft (equal), writing – review and editing (equal). **Shawn M. Wilder:** conceptualization (equal), data curation (equal), formal analysis (equal), funding acquisition (lead), investigation (equal), methodology (equal), resources (equal), validation (equal), visualization (equal), writing – original draft (equal), writing – review and editing (equal).

## Funding

S.M.W. was funded through Budget Division, NSF IOS‐2420366.

## Conflicts of Interest

The authors declare no conflicts of interest.

## Supporting information


**Figure S1:** Seasonal change in C concentrations of adult female *Larinioides cornutus* spiders in 2024. Points represent individual spiders collected on each Julian day. The solid line shows the fitted generalized linear model (GLM; Gamma distribution with log link), and the shaded ribbon indicates the 95% confidence interval. Julian days correspond to seven collection trials from mid‐June through early October.
**Figure S2:** Seasonal change in N concentrations of adult female *Larinioides cornutus* spiders in 2024. Points represent individual spiders collected on each Julian day. The solid line shows the fitted generalized linear model (GLM; Gamma distribution with log link), and the shaded ribbon indicates the 95% confidence interval. Julian days correspond to seven collection trials from mid‐June through early October.
**Figure S3:** Seasonal change in B concentrations of adult female *Larinioides cornutus* spiders in 2024. Points represent individual spiders collected on each Julian day. The solid line shows the fitted generalized linear model (GLM; Gamma distribution with log link), and the shaded ribbon indicates the 95% confidence interval. Julian days correspond to seven collection trials from mid‐June through early October.
**Figure S4:** Seasonal change in Ba concentrations of adult female *Larinioides cornutus* spiders in 2024. Points represent individual spiders collected on each Julian day. The solid line shows the fitted generalized linear model (GLM; Gamma distribution with log link), and the shaded ribbon indicates the 95% confidence interval. Julian days correspond to seven collection trials from mid‐June through early October.
**Figure S5:** Seasonal change in C concentrations of adult female *L. cornutus* spiders in 2024. Points represent individual spiders collected on each Julian day. The solid line shows the fitted generalized linear model (GLM; Gamma distribution with log link), and the shaded ribbon indicates the 95% confidence interval. Julian days correspond to seven collection trials from mid‐June through early October.
**Figure S6:** Seasonal change in Ca concentrations of adult female *Larinioides cornutus* spiders in 2024. Points represent individual spiders collected on each Julian day. The solid line shows the fitted generalized linear model (GLM; Gamma distribution with log link), and the shaded ribbon indicates the 95% confidence interval. Julian days correspond to seven collection trials from mid‐June through early October.
**Figure S7:** Seasonal variation in whole‐body Cr concentrations of adult female *L. cornutus* spiders in 2024. Points represent individual spiders collected on each Julian day. The solid line shows the fitted generalized linear model (GLM; Gamma distribution with log link), and the shaded ribbon indicates the 95% confidence interval. Julian days correspond to seven collection trials from mid‐June through early October. A quadratic model was selected based on AIC, with the dashed vertical line indicating the Julian day corresponding to the maximum predicted Cr concentration.
**Figure S8:** Seasonal change in Cu concentrations of adult female *L. cornutus* spiders in 2024. Points represent individual spiders collected on each Julian day. The solid line shows the fitted generalized linear model (GLM; Gamma distribution with log link), and the shaded ribbon indicates the 95% confidence interval. Julian days correspond to seven collection trials from mid‐June through early October.
**Figure S9:** Seasonal change in Fe concentrations of adult female *Larinioides cornutus* spiders in 2024. Points represent individual spiders collected on each Julian day. The solid line shows the fitted generalized linear model (GLM; Gamma distribution with log link), and the shaded ribbon indicates the 95% confidence interval. Julian days correspond to seven collection trials from mid‐June through early October.
**Figure S10:** Seasonal variation in whole‐body Mg concentrations of adult female *L. cornutus* spiders in 2024. Points represent individual spiders collected on each Julian day. The solid line shows the fitted generalized linear model (GLM; Gamma distribution with log link), and the shaded ribbon indicates the 95% confidence interval. Julian days correspond to seven collection trials from mid‐June through early October. A quadratic model was selected based on AIC, with the dashed vertical line indicating the Julian day corresponding to the maximum predicted Mg concentration.
**Figure S11:** Seasonal change in Zn concentrations of adult female *Larinioides cornutus* spiders in 2024. Points represent individual spiders collected on each Julian day. The solid line shows the fitted generalized linear model (GLM; Gamma distribution with log link), and the shaded ribbon indicates the 95% confidence interval. Julian days correspond to seven collection trials from mid‐June through early October.

## Data Availability

The data and R code supporting the findings are available at https://zenodo.org/records/21043997.

## References

[ece374385-bib-0001] Aitchison, J. 1986. “The Statistical Analysis of Compositional Data.” Journal of the Royal Statistical Society. Series B, Methodological 44, no. 2: 139–160. 10.1111/j.2517-6161.1982.tb01195.x.

[ece374385-bib-0002] Akaike, H. 1974. “A New Look at the Statistical Model Identification.” IEEE Transactions on Automatic Control 19, no. 6: 716–723. 10.1109/TAC.1974.1100705.

[ece374385-bib-0003] Baden, S. P. , and S. P. Eriksson . 2006. “Role, Routes, and Effects of Manganese in Crustaceans.” Oceanography and Marine Biology: An Annual Review 44: 61–83.

[ece374385-bib-0004] Barnes, L. C. 2010. “Contribution of Spider Resource Use to Ecosystem Nutrient Flow and Function.” Unpublished Doctoral Thesis. Oklahoma State University. https://www.proquest.com/dissertations‐theses/contribution‐spider‐resource‐use‐ecosystem/docview/2195467138/se‐2.

[ece374385-bib-0005] Barret, K. , B. W. Anderson , A. D. Wait , L. L. Grismer , A. G. Polis , and D. M. Rose . 2005. “Marine Subsidies Alter the Diet and Abundance of Insular and Coastal Lizard Populations.” Oikos 109: 145–153. 10.1111/j.0030-1299.2005.13728.x.

[ece374385-bib-0006] Bartz, W. , M. Górka , J. Rybak , R. Rutkowski , and A. Stojanowska . 2021. “The Assessment of Effectiveness of SEM‐EDX and ICP‐MS Methods in the Process of Determining the Mineralogical and Geochemical Composition of Particulate Matter Deposited on Spider Webs.” Chemosphere 278: 130454. 10.1016/j.chemosphere.2021.130454. 34126686

[ece374385-bib-0007] Beaubien, G. B. , C. I. Olson , A. C. Todd , and R. R. Otter . 2020. “The Spider Exposure Pathway and the Potential Risk to Arachnivorous Birds.” Environmental Toxicology and Chemistry 39, no. 11: 2314–2324. 10.1002/etc.4848. 32790212

[ece374385-bib-0008] Bozich, J. , M. Hang , R. Hamers , and R. Klaper . 2019. “Core Chemistry Influences the Toxicity of Multicomponent Metal Oxide Nanomaterials, Lithium Nickel Manganese Cobalt Oxide, and Lithium Cobalt Oxide to *Daphnia magna* .” Environmental Toxicology and Chemistry 36, no. 9: 2493–2502. 10.1002/etc.3791. 28295556

[ece374385-bib-0009] Brannon, A. C. , and K. R. Rao . 1979. “Barium, Strontium and Calcium Levels in the Exoskeleton, Hepatopancreas and Abdominal Muscle of the Grass Shrimp, *Palaemonetes pugio* : Relation to Molting and Exposure to Barite.” Comparative Biochemistry and Physiology Part A: Physiology 63, no. 2: 261–274. 10.1016/0300-9629(79)90158-0.

[ece374385-bib-0010] Bump, K. J. , O. R. Peterson , and A. J. Vucetich . 2009. “Wolves Modulate Soil Nutrient Heterogeneity and Foliar Nitrogen by Configuring the Distribution of Ungulate Carcasses.” Ecology 90, no. 11: 3159–3167. 10.1890/09-0292.1. 19967871

[ece374385-bib-0011] Chen, W. , P. Zhang , L. Ye , et al. 2024. “Concentration‐Dependent Effects of Lithium on *Daphnia magna* : Life‐History Profiles and Integrated Biomarker Response Implementation.” Science of the Total Environment 914: 169866. 10.1016/j.scitotenv.2023.169866. 38190914

[ece374385-bib-0012] Conti, E. , G. Costa , G. Liberatori , et al. 2018. “ *Ariadna* Spiders as Bioindicator of Heavy Elements Contamination in the Central Namib Desert.” Ecological Indicators 95: 663–672. 10.1016/j.ecolind.2018.08.014.

[ece374385-bib-0013] DeBenedictis, C. A. , A. Raab , E. Ducie , S. Howley , J. Feldmann , and A. M. Grabrucker . 2020. “Concentrations of Essential Trace Metals in the Brain of Animal Species—A Comparative Study.” Brain Sciences 10, no. 7: 460. 10.3390/brainsci10070460. PMC7407190 32709155

[ece374385-bib-0014] Dennis, E. B. , M. Kéry , B. J. T. Morgan , A. Coray , M. Schaub , and B. Baur . 2021. “Integrated Modelling of Insect Population Dynamics at Two Temporal Scales.” Ecological Modelling 441: 109408. 10.1016/j.ecolmodel.2020.109408.

[ece374385-bib-0015] Elser, J. J. , K. Acharya , J. Cotner , et al. 2003. “Growth Rate‐Stoichiometry Couplings in Diverse Biota.” Ecology Letters 6, no. 10: 936–943. 10.1046/j.1461-0248.2003.00518.x.

[ece374385-bib-0016] Elser, J. J. , D. R. Dobberfuhl , N. A. Mackay , and J. H. Schampel . 1996. “Organism Size, Life History, and N:P Stoichiometry: Toward a Unified View of Cellular and Ecosystem Processes.” Bioscience 46, no. 9: 674–684. 10.2307/1312897.

[ece374385-bib-0017] Elser, J. J. , M. Kyle , J. Learned , M. L. McCrackin , A. Peace , and L. Steger . 2016. “Life on the Stoichiometric Knife‐Edge: Effects of High and Low Food C:P on Growth, Feeding, and Respiration in Three Daphnia Species.” Inland Waters 6, no. 2: 136–146. 10.5268/IW-6.2.908.

[ece374385-bib-0018] Elser, J. J. , and J. Urabe . 1999. “The Stoichiometry of Consumer‐Driven Nutrient Recycling: Theory, Observations, and Consequences.” Ecology 80, no. 3: 735–751. 10.1890/0012-9658(1999)080[0735:TSOCDN]2.0.CO;2.

[ece374385-bib-0019] Evans‐White, M. A. , R. S. Stelzer , and G. A. Lamberti . 2005. “Taxonomic and Regional Patterns in Benthic Macroinvertebrate Elemental Composition in Streams.” Freshwater Biology 50: 1786–1799. 10.1111/j.1365-2427.2005.01455.x.

[ece374385-bib-0020] Fagan, W. F. , E. Siemann , C. Mitter , et al. 2002. “Nitrogen in Insects: Implications for Trophic Complexity and Species Diversification.” American Naturalist 160, no. 6: 784–802. 10.1086/343879. 18707465

[ece374385-bib-0021] Filipiak, M. 2016. “Pollen Stoichiometry May Influence Detrital Terrestrial and Aquatic Food Webs.” Frontiers in Ecology and Evolution 4: 138. 10.3389/fevo.2016.00138.

[ece374385-bib-0023] Filipiak, M. , and Z. M. Filipiak . 2022. “Application of Ionomics and Ecological Stoichiometry in Conservation Biology: Nutrient Demand and Supply in a Changing Environment.” Biological Conservation 272: 109622. 10.1016/j.biocon.2022.109622.

[ece374385-bib-0024] Filipiak, M. , and J. Weiner . 2017. “Nutritional Dynamics During the Development of Xylophagous Beetles Related to Changes in the Stoichiometry of 11 Elements.” Physiological Entomology 42, no. 1: 73–84. 10.1111/phen.12168.

[ece374385-bib-0025] Filipiak, M. , M. Woyciechowski , and M. Czarnoleski . 2021. “Stoichiometric Niche, Nutrient Partitioning and Resource Allocation in Solitary Bee Are Sex‐Specific and Phosphorous Is Allocated Mainly to the Cocoon.” Scientific Reports 11: 652. 10.1038/s41598-020-79647-7. PMC7804283 33436811

[ece374385-bib-0026] Foelix, R. F. 2011. Biology of Spiders. 3rd ed. Oxford University Press.

[ece374385-bib-0030] Gunnarsson, B. 2007. “Bird Predation on Spiders: Ecological Mechanisms and Evolutionary Consequences.” Journal of Arachnology 35, no. 3: 509–529. 10.1636/RT07-64.1.

[ece374385-bib-0031] Harrison, S. , A. Hastings , and D. R. Strong . 2005. “Spatial and Temporal Dynamics of Insect Outbreaks in a Complex Multitrophic System: Tussock Moths, Ghost Moths, and Their Natural Enemies on Bush Lupines.” Annales Zoologici Fennici 42: 409–419. https://www.jstor.org/stable/23735886.

[ece374385-bib-0032] Hawlena, D. , S. M. Strickland , A. M. Bradford , and J. O. Schmitz . 2012. “Fear of Predation Slows Plant‐Litter Decomposition.” Science 336, no. 6087: 1434–1438. 10.1126/science.1220097. 22700928

[ece374385-bib-0033] Hendrickx, F. , J. P. Maelfait , and F. Langenbick . 2003. “Absence of Cadmium Excretion and High Assimilation Result in Cadmium Biomagnification in a Wolf Spider.” Ecotoxicology and Environmental Safety 55, no. 3: 287–292. 10.1016/S0147-6513(02)00129-X. 12798762

[ece374385-bib-0034] Herzog, C. , J. T. Reeves , Y. Ipek , A. Jilling , D. Hawlena , and S. M. Wilder . 2023. “Multi‐Elemental Consumer‐Driven Nutrient Cycling When Predators Feed on Different Prey.” Oecologia 202, no. 4: 1–14. 10.1007/s00442-023-05431-9. 37552361

[ece374385-bib-0035] Herzog, C. , J. T. Reeves , and S. M. Wilder . 2025. “Testing for Differences in Consumer‐Based Nutrient Cycling Between Male and Female Wolf Spiders ( *Hogna carolinensis* ).” Ecology and Evolution 15, no. 12: e72640. 10.1002/ece3.72640. PMC12679667 41356512

[ece374385-bib-0036] Hilderbrand, G. , T. Hanley , and C. Robbins . 1999. “Role of Brown Bears ( *Ursus arctos* ) in the Flow of Marine Nitrogen Into a Terrestrial Ecosystem.” Oecologia 121: 546–550. 10.1007/s004420050961. 28308364

[ece374385-bib-0037] Hodkinson, D. I. , J. S. Coulson , J. Harrison , and R. N. Webb . 2001. “What a Wonderful Web They Weave: Spiders, Nutrient Capture, and Early Ecosystem Development in the High Arctic—Some Counter‐Intuitive Ideas on Community Assembly.” Oikos 95, no. 2: 349–352. 10.1034/j.1600-0706.2001.950217.x.

[ece374385-bib-0038] Ingenbleek, Y. , and V. R. Young . 2004. “The Essentiality of Sulfur Is Closely Related to Nitrogen Metabolism: A Clue to Hyperhomocysteinaemia.” Nutrition Research Reviews 2: 135–151. 10.1079/nrr200489. 19079922

[ece374385-bib-0039] Jensen, A. N. , and L. T. Jensen . 2014. “Chapter 1: Manganese Transport, Trafficking, and Function in Invertebrates.” In Manganese in Health and Disease, edited by L. Costa and M. Aschner , 1–33. Royal Society of Chemistry.

[ece374385-bib-0040] Jung, M. P. , and J. H. Lee . 2012. “Bioaccumulation of Heavy Metals in the Wolf Spider, *Pardosa astrigera* L. Koch (Araneae: Lycosidae).” Environmental Monitoring and Assessment 184: 1773–1779. 10.1007/s10661-011-2077-8. 21544498

[ece374385-bib-0041] Kaspari, M. 2021. “The Invisible Hand of the Periodic Table: How Micronutrients Shape Ecology.” Annual Review of Ecology, Evolution, and Systematics 52: 199–219. 10.1146/annurev-ecolsys-012021-090118.

[ece374385-bib-0042] Katsikini, M. 2016. “Detailed Spectroscopic Study of the Role of Br and Sr in Coloured Parts of the *Callinectes sapidus* Crab Claw.” Journal of Structural Biology 195, no. 1: 1–10. 10.1016/j.jsb.2016.05.006. 27183904

[ece374385-bib-0044] Kitchell, J. F. , R. V. O'Neill , D. Webb , et al. 1979. “Consumer Regulation of Nutrient Cycling.” Bioscience 29: 28–34. 10.2307/1307570.

[ece374385-bib-0045] Kleinteich, A. , S. M. Wilder , and J. M. Schneider . 2014. “Contributions of Juvenile and Adult Diet to the Lifetime Reproductive Success and Lifespan of a Spider.” Oikos 124, no. 2: 130–138. 10.1111/oik.01421.

[ece374385-bib-0047] Leal, M. C. , O. Seehausen , and B. Matthews . 2017. “The Ecology and Evolution of Stoichiometric Phenotypes.” Trends in Ecology & Evolution 32: 108–117. 10.1016/j.tree.2016.11.006. 28017452

[ece374385-bib-0048] Ludwig, L. , M. A. Barbour , J. Guevara , L. Avilés , and A. L. González . 2018. “Caught in the Web: Spider Web Architecture Affects Prey Specialization and Spider‐Prey Stoichiometric Relationships.” Ecology and Evolution 8, no. 13: 6449–6462. 10.1002/ece3.4028. PMC6053566 30038747

[ece374385-bib-0049] Marelja, Z. , S. Leimkühler , and F. Missirlis . 2018. “Iron Sulfur and Molybdenum Cofactor Enzymes Regulate the *Drosophila* Life Cycle by Controlling Cell Metabolism.” Frontiers in Physiology 9: 50. 10.3389/fphys.2018.00050. PMC5817353 29491838

[ece374385-bib-0050] Mwangi, J. N. , N. Wang , A. Ritts , J. L. Kunz , C. G. Ingersoll , and H. L. B. Deng . 2011. “Toxicity of Silicon Carbide Nanowires Sediment‐Dwelling Invertebrates in Water or Sediment Exposures.” Environmental Toxicology and Chemistry 30, no. 4: 981–987. 10.1002/etc.467 21305577

[ece374385-bib-0051] Nentwig, W. 2012. Ecophysiology of Spiders. Springer‐Verlag.

[ece374385-bib-0052] Nyffeler, M. , and K. Birkhofer . 2017. “An Estimated 400–800 Million Tons of Prey Are Annually Killed by the Global Spider Community.” Science of Nature 104: 30. 10.1007/s00114-017-1440-1. PMC5348567 28289774

[ece374385-bib-0053] Nyffeler, M. , E. J. Olson , and W. O. C. Symondson . 2016. “Plant‐Eating by Spiders.” Journal of Arachnology 44: 14–27. https://www.jstor.org/stable/24717357.

[ece374385-bib-0054] Pankau, C. , J. Nadolski , H. Tanner , et al. 2022. “Examining the Effect of Manganese on Physiological Processes: Invertebrate Models.” Comparative Biochemistry and Physiology Part C: Toxicology & Pharmacology 251: 109209. 10.1016/j.cbpc.2021.109209. PMC8922992 34628058

[ece374385-bib-0055] Peters, S. C. , R. Lockwood , C. E. Williamson , and J. E. Saros . 2008. “Using Elemental Ratios of Calcium and Strontium to Track Calcium Availability in the Freshwater Zooplankton *Daphnia pulicaria* .” Journal of Geophysical Research: Biogeosciences 113, no. G4: 2008JG000782. 10.1029/2008JG000782.

[ece374385-bib-0056] Peterson, L. R. , V. Trivett , A. J. M. Baker , C. Aguiar , and A. J. Pollard . 2003. “Spread of Metals Through an Invertebrate Food Chain as Influenced by a Plant That Hyperaccumulates Nickel.” Chemoecology 13: 103–108. 10.1007/s00049-003-0234-4.

[ece374385-bib-0057] Prater, C. , P. M. Bumpers , L. M. Demi , A. D. Rosemond , and P. D. Jeyasingh . 2020. “Differential Responses of Macroinvertebrate Ionomes Across Experimental N:P Gradients in Detritus‐Based Headwater Streams.” Oecologia 193: 981–993. 10.1007/s00442-020-04720-x. PMC7458898 32740731

[ece374385-bib-0058] Reeves, J. T. , S. D. Fuhlendorf , C. A. Davis , and S. M. Wilder . 2021. “Arthropod Prey Vary Among Orders in Their Nutrient and Exoskeleton Content.” Ecology and Evolution 11: 17774–17785. 10.1002/ece3.8280. PMC8717265 35003638

[ece374385-bib-0059] Reeves, J. T. , C. Herzog , C. Barnes , C. Davis , S. D. Fuhlendorf , and S. M. Wilder . 2023. “Variation Among Arthropod Taxa in the Amino Acid Content of Exoskeleton and Digestible Tissue.” Ecology and Evolution 13, no. 7: e10348. 10.1002/ece3.10348. PMC10365971 37496760

[ece374385-bib-0060] Romero, G. Q. , P. Mazzafera , J. Vasconcellos‐Neto , and P. C. Trivelin . 2006. “Bromeliad‐Living Spiders Improve Host Plant Nutrition and Growth.” Ecology 87, no. 4: 803–808. 10.1890/0012-9658(2006)87[803:BSIHPN]2.0.CO;2. 16676522

[ece374385-bib-0062] Sagi, N. , and D. Hawlena . 2021. “Arthropods as the Engine of Nutrient Cycling in Arid Ecosystems.” Insects 12, no. 8: 726. 10.3390/insects12080726. PMC8397162 34442292

[ece374385-bib-0063] Schaller, J. , and E. Struyf . 2013. “Silicon Controls Microbial Decay and Nutrient Release of Grass Litter During Aquatic Decomposition.” Hydrobiologia 709: 201–212. 10.1007/s10750-013-1449-1.

[ece374385-bib-0064] Schmitz, O. J. , D. Hawlena , and G. C. Trussell . 2010. “Predator Control of Ecosystem Nutrient Dynamics.” Ecology Letters 13, no. 10: 1199–1209. 10.1111/j.1461-0248.2010.01511.x. 20602626

[ece374385-bib-0065] Schmitz, O. J. , P. A. Raymond , J. A. Estes , et al. 2014. “Animating the Carbon Cycle.” Ecosystems 17: 344–359. 10.1007/s10021-013-9715-7.

[ece374385-bib-0066] Schmitz, O. J. , C. C. Wilmers , S. J. Leroux , et al. 2018. “Animals and the Zoogeochemistry of the Carbon Cycle.” Science 362, no. 6419: eaar3213. 10.1126/science.aar3213. 30523083

[ece374385-bib-0067] Sobcyzk, L. , M. Filipiak , and M. Czarnoleski . 2020. “Sexual Dimorphism in the Multielemental Stoichiometric Phenotypes and Stoichiometric Niches of Spiders.” Insects 11, no. 8: 484. 10.3390/insects11080484. PMC7469175 32751585

[ece374385-bib-0068] Sterner, R. W. , and J. J. Elser . 2002. Ecological Stoichiometry: The Biology of Elements From Molecules to the Biosphere. Princeton University Press.

[ece374385-bib-0069] Stojanowska, A. , J. Rybak , M. Bożym , T. Olszowski , and J. S. Bihałowicz . 2020. “Spider Webs as Bioindicators of Heavy Metals: A Comparison Study in the Vicinity of a Copper Smelter (Poland).” Sustainability 12, no. 19: 8066. 10.3390/su12198066.

[ece374385-bib-0070] Thibon, F. , L. Weppe , C. Churlaud , et al. 2023. “Lithium Isotopes in Marine Food Webs: Effect of Ecological and Environmental Parameters.” Frontiers in Environmental Chemistry 3: 1060651. 10.3389/fenvc.2022.1060651.

[ece374385-bib-0071] Thibon, F. , L. Weppe , N. Vigier , et al. 2021. “Large‐Scale Survey of Lithium Concentrations in Marine Organisms.” Science of the Total Environment 751: 141453. 10.1016/j.scitotenv.2020.141453. 32882547

[ece374385-bib-0072] Trubl, P. , and J. C. Johnson . 2019. “Ecological Stoichiometry of the Black Widow Spider and Its Prey From Desert, Urban and Laboratory Populations.” Journal of Arid Environments 163: 18–25. 10.1016/j.jaridenv.2018.12.002.

[ece374385-bib-0073] Vanni, M. J. 2002. “Nutrient Cycling by Animals in Freshwater Ecosystems.” Annual Review of Ecology, Evolution, and Systematics 33: 341–370. 10.1146/annurev.ecolsys.33.010802.150519.

[ece374385-bib-0074] Vanni, M. J. , A. S. Flecker , J. M. Hood , and J. L. Headworth . 2002. “Stoichiometry of Nutrient Recycling by Vertebrates in a Tropical Stream: Linking Species Identity and Ecosystem Processes.” Ecology Letters 5: 285–293. 10.1046/j.1461-0248.2002.00314.x.

[ece374385-bib-0075] Welti, E. A. R. , N. J. Sanders , K. M. De Beurs , and M. Kaspari . 2019. “A Distributed Experiment Demonstrates Widespread Sodium Limitation in Grassland Food Webs.” Ecology 100, no. 3: e02600. 10.1002/ecy.2600. 30726560

[ece374385-bib-0076] Wiggins, W. , and S. M. Wilder . 2022. “Carbohydrates Complement High‐Protein Diets to Maximize the Growth of an Actively Hunting Predator.” Ecology and Evolution 12, no. 8: e9150. 10.1002/ece3.9150. PMC9336175 35919395

[ece374385-bib-0077] Wilder, S. M. , C. L. Barnes , G. Smith , N. H. Youssef , and D. Hawlena . 2025. “Spider Waste Enhances Soil Nutrient Content, Soil Respiration, and Plant Growth.” Functional Ecology 39: 140–153. 10.1111/1365-2435.14691.

[ece374385-bib-0078] Wilder, S. M. , C. Herzog , J. T. Reeves , O. Knowles , and J. P. Cuff . 2025. “Bridging Digestive Physiology and Ecology for a More Integrative Understanding of Invertebrate Predators.” Journal of Experimental Biology 228, no. 14: jeb249697. 10.1242/jeb.249697. 40642959

[ece374385-bib-0079] Wilder, S. M. , and J. M. Schneider . 2017. “Micronutrient Consumption by Female *Argiope bruennichi* Affects Offspring Survival.” Journal of Insect Physiology 100: 128–132. 10.1016/j.jinsphys.2017.06.007. 28614727

[ece374385-bib-0080] Yamarnura, K. , M. Yokozawa , M. Nishimori , Y. Ueda , and T. Yokosuka . 2006. “How to Analyze Long‐Term Insect Population Dynamics Under Climate Change: 50‐Year Data of Three Insect Pests in Paddy Fields.” Population Ecology 48: 31–48. 10.1007/s10144-005-0239-7.

[ece374385-bib-0081] Zhang, B. , H. Chen , M. Deng , et al. 2023. “Multidimensional Stoichiometric Mismatch Explains Differences in Detritivore Biomass Across Three Forest Types.” Journal of Animal Ecology 92: 454–465. 10.1111/1365-2656.13859. 36477808

[ece374385-bib-0082] Zhou, L. , and S. A. J. Declerck . 2019. “Herbivore Consumers Face Different Challenges Along Opposite Sides of the Stoichiometric Knife‐Edge.” Ecology Letters 22: 2018–2027. 10.1111/ele.13386. PMC6900088 31512359

