# Supplementary material for: Seasonal Variation in Whole‐Body Elemental Concentrations and Excreta Production of a Riparian Orb‐Weaving Spider ( Larinioides cornutus )
Source: Ecol Evol. 2026 Sep 26;16(9):e74385. doi: 10.1002/ece3.74385 (PMC13615885; doi:10.1002/ece3.74385)
Supplement: Supplementary file 1 [file ECE3-16-e74385-s001.docx]

**Seasonal Trends in Excreta C and N Concentrations**

Figure S1: Seasonal change in C concentrations of adult female *Larinioides cornutus* spiders in 2024. Points represent individual spiders collected on each Julian day. The solid line shows the fitted generalized linear model (GLM; Gamma distribution with log link), and the shaded ribbon indicates the 95% confidence interval. Julian days correspond to seven collection trials from mid-June through early October.

Figure S2: Seasonal change in N concentrations of adult female *Larinioides cornutus* spiders in 2024. Points represent individual spiders collected on each Julian day. The solid line shows the fitted generalized linear model (GLM; Gamma distribution with log link), and the shaded ribbon indicates the 95% confidence interval. Julian days correspond to seven collection trials from mid-June through early October.

**Seasonal Trends in Whole Body Elemental Concentrations**

Figure S3: Seasonal change in B concentrations of adult female *Larinioides cornutus* spiders in 2024. Points represent individual spiders collected on each Julian day. The solid line shows the fitted generalized linear model (GLM; Gamma distribution with log link), and the shaded ribbon indicates the 95% confidence interval. Julian days correspond to seven collection trials from mid-June through early October.

Figure S4: Seasonal change in Ba concentrations of adult female *Larinioides cornutus* spiders in 2024. Points represent individual spiders collected on each Julian day. The solid line shows the fitted generalized linear model (GLM; Gamma distribution with log link), and the shaded ribbon indicates the 95% confidence interval. Julian days correspond to seven collection trials from mid-June through early October.

Figure S5: Seasonal change in C concentrations of adult female *L. cornutus* spiders in 2024. Points represent individual spiders collected on each Julian day. The solid line shows the fitted generalized linear model (GLM; Gamma distribution with log link), and the shaded ribbon indicates the 95% confidence interval. Julian days correspond to seven collection trials from mid-June through early October.

Figure S6: Seasonal change in Ca concentrations of adult female *Larinioides cornutus* spiders in 2024. Points represent individual spiders collected on each Julian day. The solid line shows the fitted generalized linear model (GLM; Gamma distribution with log link), and the shaded ribbon indicates the 95% confidence interval. Julian days correspond to seven collection trials from mid-June through early October.

Figure S7: Seasonal variation in whole-body Cr concentrations of adult female *L. cornutus* spiders in 2024. Points represent individual spiders collected on each Julian day. The solid line shows the fitted generalized linear model (GLM; Gamma distribution with log link), and the shaded ribbon indicates the 95% confidence interval. Julian days correspond to seven collection trials from mid-June through early October. A quadratic model was selected based on AIC, with the dashed vertical line indicating the Julian day corresponding to the maximum predicted Cr concentration.

Figure S8: Seasonal change in Cu concentrations of adult female *L. cornutus* spiders in 2024. Points represent individual spiders collected on each Julian day. The solid line shows the fitted generalized linear model (GLM; Gamma distribution with log link), and the shaded ribbon indicates the 95% confidence interval. Julian days correspond to seven collection trials from mid-June through early October.

Figure S9: Seasonal change in Fe concentrations of adult female *Larinioides cornutus* spiders in 2024. Points represent individual spiders collected on each Julian day. The solid line shows the fitted generalized linear model (GLM; Gamma distribution with log link), and the shaded ribbon indicates the 95% confidence interval. Julian days correspond to seven collection trials from mid-June through early October.

Figure S10: Seasonal variation in whole-body Mg concentrations of adult female *L. cornutus* spiders in 2024. Points represent individual spiders collected on each Julian day. The solid line shows the fitted generalized linear model (GLM; Gamma distribution with log link), and the shaded ribbon indicates the 95% confidence interval. Julian days correspond to seven collection trials from mid-June through early October. A quadratic model was selected based on AIC, with the dashed vertical line indicating the Julian day corresponding to the maximum predicted Mg concentration.

Figure S11: Seasonal change in Zn concentrations of adult female *Larinioides cornutus* spiders in 2024. Points represent individual spiders collected on each Julian day. The solid line shows the fitted generalized linear model (GLM; Gamma distribution with log link), and the shaded ribbon indicates the 95% confidence interval. Julian days correspond to seven collection trials from mid-June through early October.
